# Clearing the Skepticism about Subclinical Hypothyroidism: Is It Beneficial to Treat Patients with Thyroid-Stimulating Hormone >4.5 and <10 mIU/L?

**DOI:** 10.1055/s-0044-1788040

**Published:** 2024-07-03

**Authors:** Hafsa Bushra, Murtaza Rashid

**Affiliations:** 1 Department of Internal Medicine, Royal Commission Hospital Jubail, Jubail, Saudi Arabia; 2 Department of Emergency Medicine, Royal Commission Hospital Jubail, Jubail, Saudi Arabia

**Keywords:** hypothyroidism, subclinical hypothyroidism, thyroid, thyroxine

## Abstract

Subclinical hypothyroidism (SCH) is a heterogeneous clinical condition ranging from asymptomatic to wide variety of clinical manifestations, which are often nonspecific. Being a common laboratory finding, clinicians often face the dilemma of whether to treat or not. Threshold of 10 mIU/L of thyroid-stimulating hormone (TSH) is often used as a cutoff limit to offer treatment. However, still, debate remains on whether to treat less than 10 mIU/L considering special clinical conditions like pregnancy. Whether SCH exists, is screening needed in asymptomatic individuals, is treating asymptomatic cases beneficial or harmful and what threshold level of TSH to be considered for treatment are all potential questions that need to be answered.

## Introduction

Subclinical hypothyroidism (SCH) is usually defined as elevated serum thyroid-stimulating hormone (TSH) levels in absence of any symptoms of hypothyroidism and a normal circulating free T4 (FT4). The feedback between TSH and FT4 is complex. A subtle decrease in FT4 levels can result in a greater rise in the corresponding TSH values. 1 Thus, an elevated TSH with a normal range FT4 might give a biochemical diagnosis of SCH. 1 A progressive fall in FT4 below the reference value can eventually lead toward overt hypothyroidism. Thus, SCH is considered by many as a part of spectrum of primary hypothyroidism with gradual progression toward overt disease.

Prevalence of SCH is around 3 to 8% in general population. 2 The prevalence is higher in females, the elderly, and in individuals with positive anti-thyroid antibodies (Ab). 2 The incidence is about 10% in the perimenopausal women, which increases up to 20% at 75 years of age and above. 3 4 A cutoff limit of TSH of 10 mIU/L is usually used to divide the mild form of SCH from the more severe ones. Some authors also categorize SCH into grade 1 (<10 mIU/L) and grade 2 (≥10 mIU/L). 1 Approximately 75% of patients of SCH fall in the category of <10 mIU/L. 4 Common etiologies that are linked with SCH include autoimmune thyroiditis, previous radiation exposure, subtotal thyroidectomy, thyroiditis, infiltrative disorders of thyroid, and drugs (lithium, amiodarone, and interferon). 5 In contrast to hypothyroidism due to iodine deficiency, the incidence of SCH is more in the iodine-sufficient areas. 6

The general consensus for the treatment is to treat all the individuals with overt hypothyroidism as well as SCH with TSH > 10 mIU/L. Similarly, there is a consensus to treat any degree of SCH in pregnant women or those contemplating pregnancy in order to avoid pregnancy-associated complications or cognitive impairment in offspring. However, in treating individuals with TSH < 10 mIU/L only on the basis of laboratory findings and associating various nonspecific symptoms with SCH, questions like long-term benefits of thyroxine treatment, any associated risks, and follow-up plans do arise.

### Exclude Causes of Thyroid-Stimulating Hormone Assay Variability Other than Subclinical Hypothyroidism

Before concluding with the diagnosis of SCH in an individual with isolated raised TSH, one needs to consider carefully other contributing factors that might cause an elevation in TSH value. Many times an isolated increase in the TSH is transient and simply repeating the test within 2 to 3 months will show normalization of the TSH value. Physiological causes of elevated TSH include diurnal variation, age, ethnicity, and genetic polymorphism ( Table 1 ). 7 8 9 10 TSH levels are also higher in cases of recovery from nonthyroidal illness, assay variability, heterophilic Ab interference with assay, thyroid hormone resistance, certain cases of central hypothyroidism, and biotin ( Table 1 ). 2 These differentials and variations should be considered and ruled out before concluding with the diagnosis of SCH. It is also observed that within the same individual TSH value varies little around a specific set point as compared with overall population-based reference range. 11 Thus, TSH result for an individual will be higher for that person but still can fall within the normal reference range used for the population. 11

**Table 1:** Causes of isolated elevation in thyroid-stimulating hormone

Causes of elevated TSH
Physiological increase
Recovery from nonthyroidal illness
Diurnal variation
Elderly
Obesity
Recovery from thyroiditis (subacute, postpartum)
Pathological increase
Autoimmune thyroiditis
Partial thyroidectomy
Suboptimal treatment of hypothyroidism
Radioiodine ablation/external radiation to head and neck
Drugs (amiodarone, lithium, interferon alpha, iodine contrast)
Others
Assay interference
Thyroid hormone resistance
Central hypothyroidism
Renal impairment

Abbreviation: TSH, thyroid-stimulating hormone.

### Rationalizing of Upper Normal Levels of Thyroid-Stimulating Hormone

Controversy surrounds the argument regarding upper normal limit of the serum TSH. Lowering the upper limit of TSH from 5 to 3 mIU/L has been proposed by some authorities. 12 The argument favoring this lower limit is due to higher rate of detection of antithyroid peroxidase Ab (anti-TPO Ab) at TSH values between 3 and 5 mIU/L and the risk of progression to overt hypothyroidism. However, lowering the upper limit to this extent will result in overdiagnosis and overtreatment in subjects with no symptoms and of no therapeutic benefits. Moreover, in the absence of anti-TPO Abs, the risk of progression to overt hypothyroidism is considered to be low. Hence, it is not useful to consider lower threshold value for diagnosis and treatment in such cases. 13

In the elderly population, the usual upper normal limit of TSH is high, thus requiring the TSH results to be adjusted according to the age. 14 In pregnancy, TSH range of 0.03 to 2.3 mIU/L is used in first trimester and upper limit of 3.5 mIU/L is used in second and third trimesters. 15 Based on some latest recommendations, a threshold level of > 20 mIU/L is used for treatment in asymptomatic nonpregnant SCH. 16

### Controversy Regarding Screening for Subclinical Hypothyroidism

Global screening for SCH has been suggested, but it still remains a debatable topic. The American Thyroid Association (ATA) recommends screening individuals starting from the age of 35, considering higher association of elevated TSH with metabolic risks like hyperlipidemia. 17 On the other hand, the U.S. Preventive Task Force has not recommended screening in asymptomatic nonpregnant individuals as there is not enough evidence to suggest screening benefits in terms of cardiovascular (CV) disease and overall morbidity and mortality. 18 The American College of Physicians suggests screening in women older than 50 years of age. 19 Considering the important implications of SCH in pregnancy, active case search and screening for SCH is recommended by all authorities in pregnant women or those anticipating pregnancy. 20

### Long-Term Complications Related to Subclinical Hypothyroidism (Thyroid-Stimulating Hormone > 4.5 and <10 mIU/L) and Effects (Benefit/Risk) of Treatment

SCH like overt hypothyroidism is often linked to heart failure (HF), 21 ischemic heart disease, 22 deranged lipid profile, 23 increase in risk of stroke, 24 memory decline, 25 depression, 26 fatigue, and poor quality of life. 27 SCH results in poor outcomes in pregnancy in form of miscarriage, placental abruption, preeclampsia, and perinatal mortality. 28 However, whether an association really exists between SCH and these symptoms and treatment with thyroxine can ameliorate these symptoms are still unclear. On the other hand, overdiagnosis and overtreatment of the SCH have resulted in widespread use of thyroxine, making it one of the most prescribed medications and has resulted in increased risk of overt or subclinical hyperthyroidism. 29

In order to understand SCH association with systemic manifestations, we need to understand long-term implications of uncontrolled SCH on various systems and the effect of thyroxine treatment in terms of reversing or improving these morbidities.

#### Cardiovascular Disease

CV abnormalities that are present in overt hypothyroidism are also identified in individuals with SCH. Studies favoring association of CV risks with SCH are more than those showing lack of association. However, whether treating SCH (TSH < 10 mIU/L) will result in reversing these CV changes is not proven and hence making the decision of treatment difficult and biased.

CV abnormalities seen in SCH include left ventricular diastolic dysfunction, 30 systolic dysfunction during exercise, and limitation in exercise capacity. 31 32 33 In a meta-analysis by Moon et al, a modest association between SCH and CV disease was observed; however, no similar association was seen in people older than 65 years of age. 34 Another prospective cohort study showed an increased risk of HF with SCH, supporting HF onset and progression in SCH ( Table 2 ). 21 TSH ≥ 7 mIU/L has been linked with increased risk of severe HF, atrial fibrillation (AF) and composite end point of ventricular assist device placement in one study. 35

**Table 2:** Role of treatment of subclinical hypothyroidism (thyroid-stimulating hormone 4.5–9.9 mIU/L) with thyroxine on various adverse effects of subclinical hypothyroidism

Adverse effect	Role of treatment with thyroxine
Coronary artery disease	Insufficient data to support benefit of treatment
Congestive heart failure	Insufficient data to support benefit of treatment
Stroke	Insufficient data to support benefit of treatment
Cognitive dysfunction	Insufficient data to support benefit of treatment
Surrogate markers of CV risk (elevated total CH, LDL-C, increased intima-media thickness, and reduced cardiac function)	Moderate benefit by reduction in total CH and LDL-C level but unclear whether this is accompanied by decreased CV events
Neuromuscular dysfunction, exercise intolerance	Insufficient data to support benefit of treatment
Progression toward overt hypothyroidism	Early treatment is beneficial before development of overt hypothyroidism (especially with positive anti-TPO Ab, goiter)

Abbreviations: Ab, antibody; CH, cholesterol; CV, cardiovascular; LDL-C, low-density lipoprotein—cholesterol; TPO, thyroid peroxidase.

Vascular abnormalities in the form of increased vascular resistance and altered vascular compliance are seen in SCH. 36 37 Increased vascular resistance leads to altered stiffness of vessels, elevated arterial pressure, and hypertension (HTN). 36 37 Increased acceleration and progression of atherosclerosis is seen in SCH, which could partially be explained by dyslipidemia, increased vascular stiffness, and diastolic HTN. 38 However, increased activity of innate immune system in the form of elevated tumor necrosis factor-α, matrix metalloproteinase-9, and nuclear factor kappa-β has also been postulated as a predisposing factor to result in advancement in atherosclerosis. 38 Another explanation for CV effects seen in SCH and overt hypothyroidism is the expression of thyroid hormone receptors on vascular and myocardial endothelial cells. 39 Thyroid hormone via its nongenomic and genomic pathway manipulates the expression of ion channels, structural and regulatory proteins, thus affecting the CV parameters as seen in these cases. Carotid artery intima-media thickness (CIMT) and coronary artery disease (CAD) incidence have been found to be increased in SCH. 40 A systematic review and meta-analysis of 29 clinical trials showed SCH association with increased CIMT and decreased flow-mediated dilatation values. 41 Meta-analysis of individual data of 11 prospective cohort studies found increased risk of fatal and nonfatal CAD events with higher TSH levels (hazard ratio [HR]: 1.00 when TSH = 4.5–6.9 mIU/L, HR: 1.17 when TSH = 7–9.9 mIU/L, and HR: 1.89 when TSH = 10–19.9mIU/L). 5

Dyslipidemia, in the form of elevated low-density lipoprotein cholesterol (LDL-C), triglycerides (TG), and apolipoprotein B occur in SCH. 23 A meta-analysis of 16 studies involving 41,931 individuals showed SCH association with elevated total cholesterol, TG, and LDL-C as compared with euthyroid individuals. 42 On the other hand, Hueston and Pearson found no association between elevated LDL-L and TG with SCH in 8,228 participants. 43 Hyland et al demonstrated no link between SCH with CAD, HF, and cerebrovascular death in patients with age 65 years and above. 44

The benefits of treatment in terms of improvement in cardiac parameters like systolic and diastolic dysfunction, systemic vascular resistance, and endothelial function are seen in SCH. 45 Meta-analysis by Nakanishi et al showed treatment with levothyroxine in SCH reduces both total as well as LDL-C. 46 A review of 16 clinical studies involving 19,020,189 participants showed reduction in CV events and improved quality of life in individuals with TSH > 10 and prevention of CV events when subjected to levothyroxine treatment. 47 Meta-analysis of 12 clinical trials showed beneficial effects of levothyroxine in SCH in terms of reduction in CIMT values and prevention in terms of atherosclerosis development. 48 A recent meta-analysis of 11 studies showed improvement in cardiac output, left ventricular ejection fraction, and E/A ratio (ratio of peak velocity blood flow due to left ventricular relaxation in early diastole to peak velocity flow in late diastole caused by atrial contraction) with levothyroxine therapy in SCH. 49 Contrary to these studies, a Danish cohort study failed to show association between thyroxine treatment and myocardial infarction except in younger patients. 50 A retrospective cohort study in cooperating 12,212 participants with SCH failed to show any evidence of benefit of levothyroxine in terms of incidence of myocardial infarction, CV death, and overall mortality. 51 Similarly TRUST trial showed no beneficial effect of thyroxine replacement in terms of fatal and nonfatal CV events in SCH group versus control. 52 Feller et al meta-analysis incorporating 21 randomized controlled trials (which included the TRUST trial as well) failed to show beneficial effect of thyroxine in terms of improvement in quality of life and thyroid-related symptoms in SCH. 53

Thus, in conclusion, the evidence of CV effects of SCH comes from many observational studies with increasing risk associated with rising TSH levels (TSH > 10 mIU/L). Data are still lacking in defining exactly which surrogate markers of CV disease (cholesterol levels, myocardial contractility, endothelial dysfunction, and intima-media carotid artery thickness) and metabolic parameters (body mass index and waist circumference) benefit more from thyroxine treatment. Diversity of results between different studies emphasizes the need for more long-term multicenter randomized trials to assess the benefits of thyroxine treatment on CV parameters in SCH and to identify which subgroup of patients (mild SCH/severe SCH) might benefit more. 54

#### Cerebrovascular Disease

Cerebrovascular disease has also been linked to SCH ( Table 2 ). A meta-analysis by Chaker et al including 47,573 participants failed to show any increased overall risk of stroke in individuals with SCH. 55 However, in the same study, an increased risk of stroke events and fatal stroke was seen in individuals with age <65 years and with higher TSH values (HR: 1.18 with TSH = 4.5–6.9 mIU/L, HR: 1.63 with TSH = 7.0–9.9 mIU/L, and HR: 1.69 with TSH =  10–19.9 mIU/L). 55

#### Renal Compromise

SCH is associated with reduction in glomerular filtration rate (GFR) and progression toward renal failure. 56 As high TSH results could be due to decreased renal clearance secondary to reduced GFR rather than thyroid dysfunction, it is difficult to define a true association between SCH and renal impairment. SCH has a negative impact on GFR in patients with established renal disease or associated conditions that lead to compromised renal status like diabetes mellitus (DM) and HTN. 57 Treating SCH can halt progression of renal impairment was studied by Adrees et al. 58 The results showed a positive impact of treatment in terms of reduced CV risk factors, increased carotid artery diameter, and normalized estimated GFR. 58 However, TSH levels of individuals in this study were higher (up to 18.8 mIU/L), making it difficult to generalize these findings in individuals with mild elevation of TSH. Another recent prospective study failed to show any association of renal impairment with SCH (TSH level < 10 mIU/L). 59

#### Dementia and Impaired Cognition

SCH is often linked to cognitive impairment and memory decline. Whether such an association exists, and treatment improves these symptoms, is a debatable subject. A meta-analysis by Pasqualetti et al including 13 studies showed high risk of cognitive impairment and dementia in individuals < 75 years of age. 25 On the other hand, a meta-analysis by Rieben et al failed to show cognitive disturbance, memory impairment, and decline of Mini-Mental State Examination in SCH patients as compared with controls ( Table 2 ). 60 Two placebo-controlled trials of treatment with thyroxine failed to show improvement in cognition in SCH. 61 62 However, there were limitations in these trials, in terms of using single TSH values, shorter treatment duration, and TSH range of 3.5 to 4.9 mIU/L, thus including many euthyroid individuals. 61 62 Recent TRUST trial failed to confirm any beneficial effect of thyroxine treatment on executive cognitive function in SCH. 52

#### Quality of Life and Other Clinical Parameters

Poor quality of life, fatigue, muscle weakness, weight gain, obesity, cold intolerance, and constipation are linked with SCH. 4 31 Symptoms are usually mild as compared with those seen with overt hypothyroidism. The obvious link between these symptoms and SCH is not very clear. Reasons could be due to difference in selection of patients, different age groups, and baseline TSH variation between different study groups. Elderly persons were found to be less symptomatic with SCH as compared with younger persons. 63 64 65 A meta-analysis by Feller et al failed to show improvement in quality of life or thyroid-related symptoms with thyroxine in SCH patients. 53

#### Potential Risks of Treatment

With an increasing trend toward screening asymptomatic individuals by TSH assay in the last two decades, there is a rise in diagnosis of SCH and prescription of thyroxine. Worldwide, thyroxine is now among the most prescribed medications. 66 As these patients have minimal elevation of TSH, unlike the overt hypothyroidism, the risk of overtreatment/undertreatment is higher. This requires more meticulous monitoring and follow-up, thus increasing cost and burden on health care system. This challenge becomes more difficult to meet in elderly patients, who are at higher risk of polypharmacy, compliance problems, drug interactions, and morbidity. Intake of thyroxine requires eating habits to be modified by taking medicine on an empty stomach and avoiding milk and iron-containing preparations. Once treatment is started, it often continues indefinitely in majority of the cases. 67 Moreover, as observed in many studies, there is no clinical improvement in patients' nonspecific symptoms with thyroxine treatment. Last and most important, these patients are exposed to the risk of subclinical or overt hyperthyroidism, increasing the risk of AF and osteoporosis, especially in the elderly. 29 68 This fact was confirmed by a retrospective cohort study, showing that with the increasing trend of thyroxine prescription for milder TSH elevation, suppressed TSH is more observed in treated population. 67

### Special Population Groups

While taking into consideration the treatment for SCH, it's worthy to consider different population-based groups separately and considering impact of SCH in these groups and benefits/risks of the treatment.

#### Pregnancy

Pregnancy is considered to be a stressful condition due to alteration in normal physiology. It requires increased production of thyroid hormones in order to maintain enough supply for the developing fetus and meet new physiological demands of the maternal body. Hypothyroidism during this period can have deleterious effects on the mother and her newborn. Human chorionic gonadotropin levels increase especially in the first trimester. Due to its cross-reactivity with TSH receptor, the level of TSH drops to an upper limit of 2.5 mIU/L. Based on this, reference ranges for TSH as suggested by ATA (2011) and Endocrine Society (2012) are 0.1 to 2.5, 0.2 to 3.0, and 0.3 to 3.5 mIU/L in first, second, and third trimesters, respectively. 15 69 However, this reference range was not found applicable in other geographical areas and ethnic origins; thus, the latest ATA (2017) guidelines recommend local population-based reference range to be used in each trimester of pregnancy instead of universal cutoff values. 70 Considering limitation and paucity of local data, ATA guidelines (2017) also suggest in situations of lack of local data, to decrease the upper limit of normal range of TSH in first trimester by 0.5 mIU/L from nonpregnant reference range, which corresponds to 4 mIU/L. 70 This reference range is applicable in first trimester with return to nonpregnancy upper reference range of TSH in second and third trimesters. 70 Thyroid auto-Abs are detected in up to 50% of pregnant women with SCH. 8

SCH in pregnancy is associated with many adverse events in terms of preterm delivery, miscarriage, placental abruption, premature rupture of membrane, and neonatal death. 71 72 73 Overall, there is conflict in the data linking obstetrical and neonatal complications with SCH. The reason for this could be due to inconsistency in risk reporting, coexisting variables, underpowered studies, and use of the dependent obstetric outcomes. 74

Treatment of SCH in pregnancy has also shown variable results in terms of benefits versus no change in outcome. A retrospective cohort study of pregnant women with SCH (TSH = 4.1–10 mIU/L) treated with thyroxine had fewer poor outcomes in terms of pregnancy loss as compared with controls. 75 A randomized controlled trial, assessing the effect of thyroxine treatment on the IQ level of offspring of pregnant women, failed to show any difference in outcome between the treatment group and placebo. 76 Meta-analysis by Yamamoto et al, assessing effects of treatment in pregnant women in terms of obstetrical (miscarriage, gestational HTN, preeclampsia, and preterm delivery) and childhood outcomes (neonatal ICU admission, birth weight, gestational age at delivery, childhood IQ, and neurodevelopment scores) failed to show any evidence of benefit. 77 All these data raise issue of conflict between individual studies and lack of consensus of management.

The latest 2017 ATA guidelines recommend treating all SCH pregnant women with replacement therapy when anti-TPO Ab is positive with TSH above pregnancy-specific reference range (strong recommendation). 70 In anti-TPO Ab positive women, with TSH > 2.5 mIU/L and below the upper limit of pregnancy-specific reference range can be offered treatment (weak recommendation, moderate-quality evidence). 70 In TPO-negative women, treatment is recommended when TSH is >10 mIU/L (strong recommendation), whereas thyroxine treatment can be considered in women with TSH above pregnancy-specific reference range and below 10 mIU/L (weak recommendation, low-quality evidence). 70 However, there are concerns raised about these recommendations in other guidelines (Italian Thyroid Association, 2017) as false-negative test for thyroid Ab can occur in pregnant women due to immune suppression. 78 Thus, SCH diagnosed prior to pregnancy or during pregnancy needs individualized treatment decisions after discussion with the patient with follow-up of thyroid function tests 4 to 6 weeks to avoid iatrogenic hyperthyroidism. 79

#### Elderly Population

The most formidable demographic challenge facing the world is aging population. Various studies have revealed an increased prevalence of SCH in the geriatrics. The most important consideration in this regard is that TSH level shifts to higher upper limits with the advancing age in normal individuals. 9 Many studies failed to prove association of SCH with CV risk and lipid abnormalities in the elderly. Gussekloo et al revealed a prolonged life span in elderly with SCH. 63 A Japanese study in the elderly reported spontaneous normalization of TSH in 53.5% participants in follow-up, whereas 7.0% progressed to overt hypothyroidism. 80 Effects of thyroxine treatment on various symptoms related to thyroid dysfunction were studied in the TRUST trial, which failed to show any improvement in symptoms with a replacement strategy. 52 Thus, the consensus stands for treating only overt hypothyroidism in the elderly population. The decision to treat or not to treat SCH in elderly should not be based on clinical judgment as symptoms are highly nonspecific and the risk of overtreatment (CV risk in the form of AF and bone damage in the form of osteoporosis) is higher. 31 81

#### Type 1 and Type 2 Diabetes Mellitus

Thyroid dysfunction is common in both type 1 and 2 DM. SCH is more prevalent in type 1 DM due to clustering of autoimmune diseases. 82 For unknown reasons, patients with type 2 DM have more than twice the risk of developing SCH as compared with the healthy population. 82 Treatment is recommended usually if TSH > 10 mIU/L. 18 At TSH level < 10 mIU/L, treatment decision need to be individualized based on the presence of associated dyslipidemia, insulin resistance, and risk of progression to overt hypothyroidism.

### Guidelines and Recommendations

Current guidelines and expert opinion recommend threshold of treatment for SCH as >10 mIU/L for adults ( Table 3 ). 1 5 18 83 84 85 Treatment is recommended at TSH of 4.5 to 9.9 mIU/L only if younger than 65 to 70 years, having symptomatic hypothyroidism, or have definite indications (like CV disease or presence of anti-TPO Ab). A recent panel suggests raising TSH limit to >20 mIU/L as a threshold for treatment. 16 However, these recommendations are not valid for women who are trying to get pregnant or are already pregnant, younger adults (≤30 years), and with severe symptoms of hypothyroidism. 16

**Table 3:** Guidance for treatment for subclinical hypothyroidism (Guidelines and Expert Opinion Summary)

Organization/guidelines/expert review	Recommendation
National Institute of Health and Care Excellence (NICE) guidelines 2019 83	TSH > 10 mIU/L Consider treatment TSH 4–10 mIU/L Age <65 y with symptoms, trial of treatment Age ≥ 65 y wait and watch
European Thyroid Association 2013 84	Age < 70 y TSH >10 mIU/L treat TSH <10 mIU/L without symptoms, observe TSH <10 mIU/L with symptoms, start trial Age > 70 y TSH < 10 mIU/L observe TSH > 10 mIU/L, consider treatment if symptoms or high CV risk
American Thyroid Association 2012 18	TSH > 10 mIU/L, consider treatment TSH < 10 mIU/L, consider treatment if symptoms suggest, positive thyroid antibody, evidence of atherosclerotic CV abnormality or heart failure.
UpToDate 2022 85	TSH <7 mIU/L Age > 65–70 y observe Age <65–70 y treat if symptoms, observe if no symptoms TSH 7–10 mIU/L Age > 65–70 y treat if symptoms, observe if no symptoms Age <65–70 y treat TSH > 10 mIU/L treat
Rapid recommendation *BMJ* 2019 16	TSH < 20 mIU/L Recommend against treatment except young adults <30 y, women who are and trying to become pregnant, or with severe symptoms TSH > 20 mIU/L treat
*JAMA* review 2019 5	Age < 65 y TSH = 4.5–6.9 mIU/L treat if symptoms of hypothyroidism, positive TPO, planning pregnancy, progressively increasing TSH, goiter TSH = 7.0–9.9 mIU/L treat to decrease risk of stroke, CAD mortality TSH ≥10 mIU/L treat Age ≥ 65 y TSH 4.5–6.9mIU/L, no treatment TSH 7.0–9.9 mIU/L, treat to decrease risk of stroke, CAD mortality TSH ≥ 10 mIU/L treat
*N Engl J Med* 2017 1	Age ≤ 70 y TSH ≥ 10 mIU/L treat Age ≥ 70 y or TSH < 10 mIU/L Treat if symptoms, positive anti-TPO, or cardiac risk factors

Abbreviations: CAD, coronary artery disease; TPO, thyroid peroxidase; TSH, thyroid-stimulating hormone.

## Conclusion

In conclusion, SCH is a heterogeneous clinical condition from being asymptomatic to a wide range of clinical manifestations and involving different population groups. Due to the different clinical settings, therapeutic decisions should be individualized and taken into consideration of concomitant diseases, risk factors for progression, and physiological conditions (pregnancy). More research and studies are highly needed to address controversial issues surrounding the treatment benefits in SCH in various population groups.
